# The association between RANK, RANKL and OPG gene polymorphisms and the risk of rheumatoid arthritis: a case-controlled study and meta-analysis

**DOI:** 10.1042/BSR20182356

**Published:** 2019-06-28

**Authors:** Haoyu Yang, Weixi Liu, Xindie Zhou, Huan Rui, Hui Zhang, Ruiping Liu

**Affiliations:** 1 Department of Orthopedics, The Affiliated Changzhou No. 2 People’s Hospital of Nanjing Medical University, Changzhou, China; 2 School of Clinical Medicine, Xuzhou Medical University, Xuzhou, China; 3 Laboratory of Biochemical Engineering, College of Pharmaceutical Engineering and Life Sciences, Changzhou University, Changzhou, China

**Keywords:** case-controlled study, polymorphism, RA, RANK, RANKL

## Abstract

The receptor activator of nuclear factor-κB (RANK) and the osteoprotegerin (OPG) cascade system have been reported to be essential in osteoclastogenesis. In recent years, several studies have investigated the association between polymorphisms of RANK, its ligand *RANKL* and *OPG* genes and the risk of rheumatoid arthritis (RA) in different populations. However, the results arising from these studies were conflicting. To determine the association between *RANK, RANKL* and *OPG* gene polymorphisms and the risk of RA. We conducted a hospital-based case-controlled study in Changzhou with 574 RA cases and 804 controls. The genotyping of *RANK* gene rs1805034 polymorphism was conducted by single base extension combined with matrix-assisted laser desorption/ionization time of flight mass spectrometry (MALDI-TOF-MS). We also undertook a meta-analysis of the literature referring to polymorphisms of *RANK, RANKL* and *OPG* genes and RA risk. This case-controlled study found that the polymorphism in the *RANK* gene rs1805034 was not related to RA risk. Stratification analyses by sex and age suggested that *RANK* gene rs1805034 polymorphism was not associated with the risk of RA among groups of male, female, age ≤ 55 and age > 55. Our meta-analysis found that the rs2277438 polymorphism in *RANKL* gene increased the risk of RA, whereas *RANK* gene rs1805034, *OPG* gene rs3102735, *OPG* gene rs2073618, *OPG* gene rs3134069 polymorphisms were not related to RA susceptibility. In conclusion, this case-controlled study and meta-analysis indicated that the *RANKL* gene rs2277438 polymorphism increased the RA risk, and that *RANK* gene rs1805034, *OPG* gene rs3102735, *OPG* gene rs2073618, *OPG* gene rs3134069 polymorphisms were not related to RA risk.

## Introduction

With progressing age, there are fundamental changes in the immune system and the propensity for abnormal immunity [1]. Individuals who are more than 50 years of age are not only more susceptible to infection and cancer, but are also at a higher risk of chronic inflammation. The process of immunosenescence is accelerated in rheumatoid arthritis (RA) [1], a systemic autoimmune disease [2] characterized by chronic progressive articular inflammation. Despite its low prevalence [3], no significant reduction in mortality has been demonstrated in different RA populations worldwide [4,5]. Multiple factors could affect the development of RA [3], and the etiology and pathogenesis of RA are not completely understood. However, several lines of observational evidence have indicated that osteoclasts and monocytic cells are key mediators of the bone loss which occurs during the course of RA. As members of the tumor necrosis factor (TNF) family, the receptor activator of nuclear factor-κB (RANK), its ligand RANKL, and osteoprotegerin (OPG, a decoy receptor of RANK) are known to have significant impacts on the central regulation of osteoclast development and activation [6,7]. A previous study identified that single nucleotide polymorphisms (SNPs) located on *RANK, RANKL* and *OPG* were associated with the presence of anti-citrullinated peptide antibody (ACPA) or erosions in RA patients [8]. Moreover, the application of anti-rheumatic drugs has been shown to modulate the expression of RANKL and OPG by the synovial tissue in RA, thus preventing cartilage and bone damage. Thus, we hypothesized that the RANK, RANKL and OPG network may play an important role in the pathogenesis of RA.

The association between the *RANK* gene, *RANKL* gene, *OPG* gene SNPs and RA susceptibility may provide new research directions for RA studies. Thus far, several studies [9–16] have explored the relationship between polymorphisms in the *RANK* and *RANKL* genes but achieved conflicting and inconclusive results. As gene pools, lifestyle, and gene–environment interactions vary between populations, we cannot expect risk to be identical across every population with respect to genotypes. Therefore, we conducted this case-controlled study to investigate the association between *RANK* gene rs1805034 polymorphism and RA susceptibility in a Chinese Han population. We also realized that a single case-controlled study may not have full statistical power and may lead to inconclusive results owing to limited sample sizes, clinical heterogeneity and different ethnic populations. Therefore, we further performed an additional meta-analysis to verify the relationship between known SNPs in the *RANK, RANKL* and *OPG* genes and RA and thus yielded more robust conclusions.

## Methods

### Study population

This hospital-based case-controlled study was approved by the Ethics Committee of the Affiliated Changzhou No.2 People’s Hospital of Nanjing Medical University and performed according to the *Declaration of Helsinki*.

In total, 574 hospitalized RA patients (427 females and 147 males) were recruited from the Affiliated Changzhou No.2 People’s Hospital of Nanjing Medical University, the Changzhou Traditional Chinese Medical Hospital and the Changzhou First Hospital. These patients were diagnosed using the criteria published by the American College of Rheumatology/European League against Rheumatism Collaborative Initiative for RA [17]. Patients of other nationalities, with other major systemic diseases, other autoimmune diseases, or a family history of autoimmune diseases were all excluded.

The 804 controls (500 females and 205 females) were patients without RA, matched for age and sex and were recruited from the same institutions during the identical period. Most of the controls were trauma patients. In order to acquire information relating to demographic data and related risk factors, each patient was interviewed personally using a pre-tested questionnaire; this was done after patients had provided written informed consent.

### Genomic DNA extraction and genotyping

Ethylenediaminetetraacetic acid (EDTA) tubes were used to store blood samples. Genomic DNA was isolated from whole blood using a QIAamp DNA blood mini kit (Qiagen, Hilden, Germany). SNPs were genotyped by a MassARRAY system (Sequenom, San Diego, California) and by matrix-assisted laser desorption/ionization time-of-flight mass spectrometry (MALDI-TOF-MS) which was performed without knowledge of patient status (case *vs.* control) to ensure the quality of genotyping, as previously described [18].

### Statistical analysis

The relationship between the studied SNPs and RA risk was accessed by calculating the odds ratio (OR) and 95% confidence intervals (CI) for five gene models (allele, dominant, recessive, homozygous, and heterozygous). Demographic characteristics and the genotypes of the studied genes were evaluated using a chi-squared (χ^2^) test (for categorical variables) or Student’s *t* test (for continuous variables). All statistical analyses were performed on SAS software package (ver. 9.1.3; SAS Institute, Cary, NC, U.S.A.) with a significance level of *P*<0.05. Hardy–Weinberg equilibrium (HWE) of the genotypes was analyzed using the goodness-of-fit χ^2^ test, to compare the observed and expected genotype frequencies among controls. To thoroughly investigate the association of SNPs in the genes of *RANK, RANKL* and *OPG* with RA, we also conducted a meta-analysis which was performed using the Stata 11.0 software (StataCorp, College Station, TX, U.S.A.).

## Results

### Clinical details of the study population

The characteristics of the study population are summarized in Table 1. Cases and controls were well matched in terms of age and sex (*P*=0.080 and *P*=0.962, respectively), and no significant differences in age and sex were observed between the RA patients and controls. The frequency distribution of the rs1805034 genotypes in the RA patients and control subjects are shown in Table 2 and conformed to the HWE in each group.

**Table 1:** Patient demographics and risk factors in RA

Variable	Cases (*n*=574)	Controls (*n*=804)	*P*
Age (years)	54.5 ± 15.1	55.7 ± 10.1	0.080
Female/male	427/147	599/205	0.962
Onset age (years)	45.6 ± 12.9		
Disease duration (years)	8.9 ± 9.2		
Treatment duration (years)	7.6 ± 7.8		
RF-positive	456 (79.4%)		
ACPA positive	300 (52.2%)		
CRP-positive	323 (56.3%)		
ESR (mm/h)	33.7 ± 25.2		
DAS28	4.3 ± 1.5		
Functional class			
I	73 (12.7%)		
II	256 (44.6%)		
III	209 (36.4%)		
IV	36 (6.3%)		

Abbreviations: CRP, C-reactive protein; ESR, erythrocyte sedimentation rate; DAS28, RA disease activity score; RF, rheumatoid factor.

**Table 2:** Logistic regression analysis of associations between rs1805034 polymorphism and the risk of RA

Genotype	Cases (*n*=574)		Controls (*n*=804)		OR (95% CI), *P*	Adjusted OR (95% CI); *P*
	*n*	%	*n*	%		
RANK rs1805034						
TC vs. TT	253/266	44.3/46.6	324/386	41.3/49.2	1.13 (0.90, 1.42), 0.280	1.13 (0.90, 1.42); 0.287
CC vs. TT	52/266	9.1/46.6	75/386	9.5/49.2	1.01 (0.68, 1.48), 0.975	0.99 (0.77, 1.27); 0.992
CC+TC vs. TT	305/266	53.4/46.6	399/386	50.8/49.2	1.11 (0.89, 1.38), 0.347	1.11 (0.89, 1.37); 0.360
CC vs. TC+TT	52/519	9.1/90.9	75/710	9.5/90.5	0.95 (0.64, 1.38), 0.780	0.94 (0.65, 1.37); 0.749
C vs. T	357/785	31.3/68.7	474/1096	30.2/69.8	1.05 (0.89, 1.24), 0.551	NA

Adjusted for age and sex. Abbreviation: NA, not available.

### Association between RANK gene rs1805034 polymorphism and RA risk

The genotypic distribution of the *RANK* gene rs1805034 polymorphism in all subjects are delineated in Table 2. Logistic regression analyses revealed that the CC genotype, or C allele carriers of the rs1805034 polymorphism, were not associated with the risk of RA (TC *vs.* TT; adjusted OR = 1.13, *P*=0.0287; CC *vs.* TT; adjusted OR = 0.99, *P*=0.992; CC+TC *vs.* TT; OR = 1.11, *P*=0.360; CC *vs.* TC+TT; adjusted OR = 0.94, *P*=0.749; C *vs.* T; OR = 1.05, *P*=0.551, Table 2). Furthermore, the effects of this SNP on RA risk were further evaluated according to age and sex; no significant association was found (Supplementary Table S1). Furthermore, no significant association was found between rs1805034 genotypes and clinical or biochemical characteristics. Finally, no significant differences were found in terms of demographic or laboratory data when compared between CC+TC and TT genotypes (Supplementary Table S2) or between CC and TC+TT genotypes (Supplementary Table S3 & Table S4).

### Meta-analysis: general characteristics of the included studies and quantitative analysis

**Figure 1:**
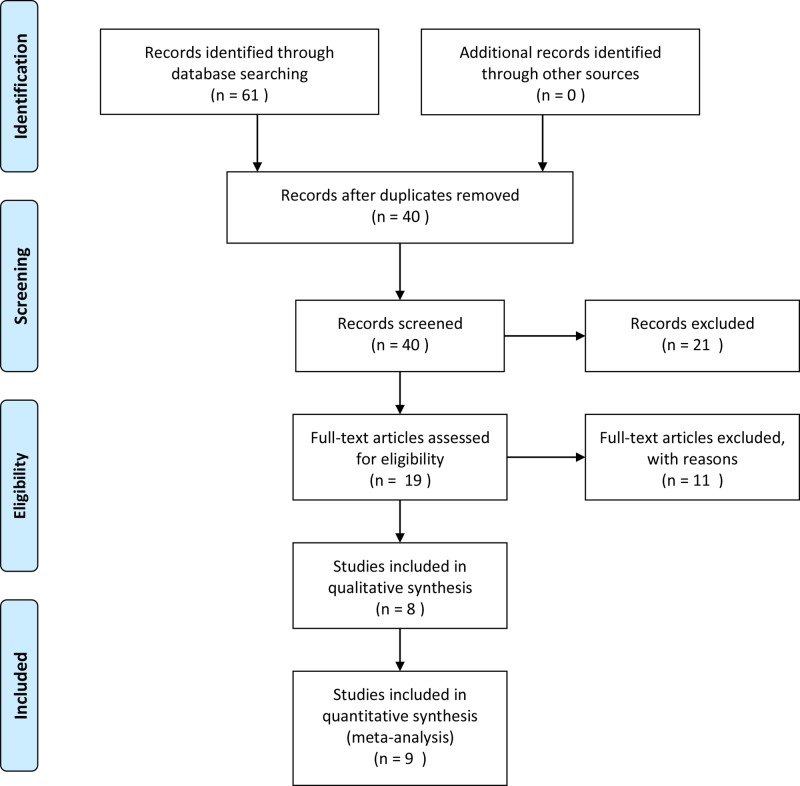
Flowchart describing how the literature search was performed and how individual studies were selected for analysis

All included studies were carefully selected and our literature review was up-to-date as of July 2018. Our selection protocol for qualified studies is presented in Figure 1. The characteristics of the studies included in this meta-analysis investigating the associations between the SNPs of *RANK, RANKL, OPG* genes and RA risk are listed in Supplementary Tables S5 and S6. Five Asian studies (including the present study) and four Caucasian studies, were identified for inclusion in this meta-analysis. The Newcastle–Ottawa Scale (NOS) scores of all included studies ranged from 5 to 7 stars, suggesting that they were studies of high methodological quality.

**Figure 2:**
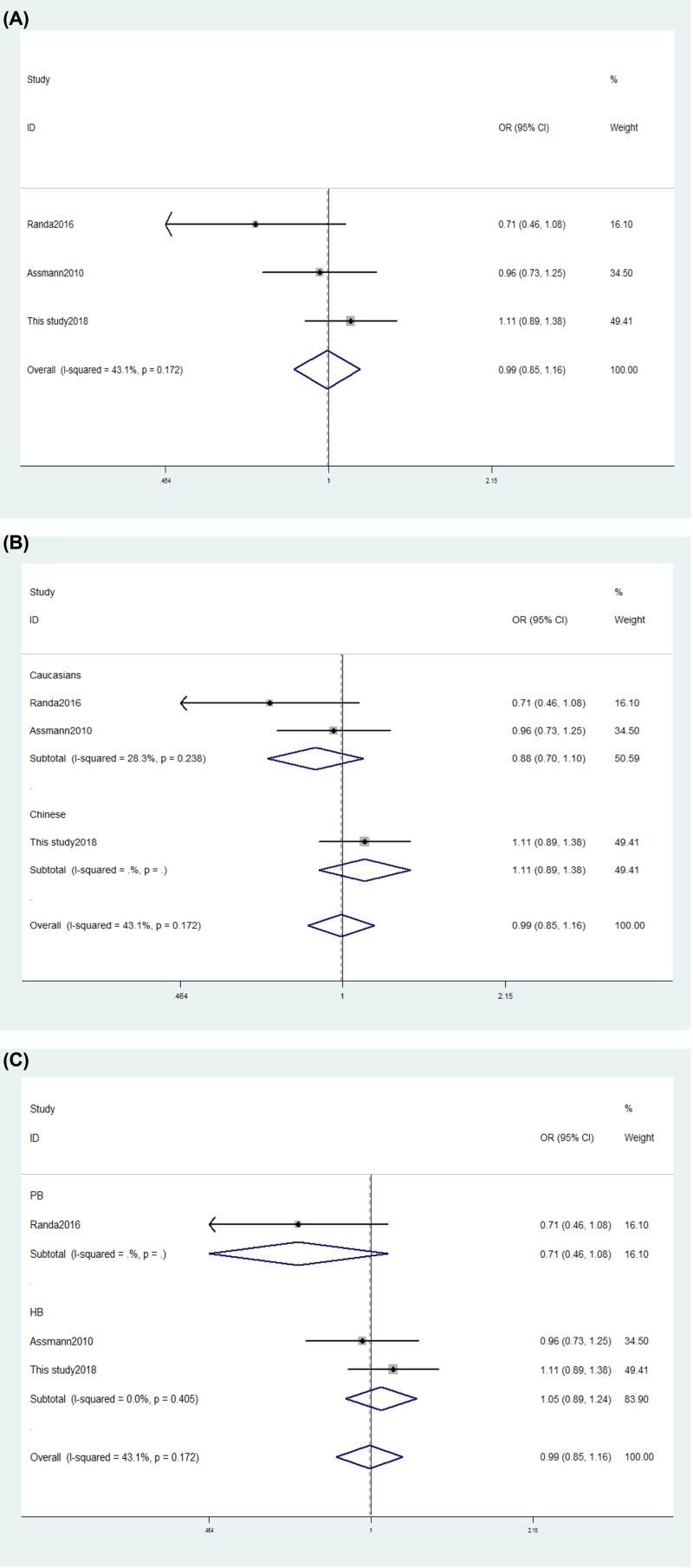
Forest plot showing the OR for association between the rs1805034 polymorphism and the risk of RA (**A**) Forest plot showing the OR for associations between the rs1805034 polymorphism and the risk of RA (CC+TC *vs.* TT). (**B**) Stratification analysis by ethnicity showing the OR for associations between the rs1805034 polymorphism and RA risk (CC+TC *vs*. TT). (**C**) Stratification analysis by SOC showing the OR for associations between the rs1805034 polymorphism and the risk of RA (CC+TC *vs.* TT).

Our meta-analysis indicated that the *RANK* rs1805034 polymorphism was not associated with the risk of RA (C *vs.* T: OR = 0.99, *P*=0.923; CC+TC *vs.* TT: OR = 0.99, *P*=0.927; CC *vs.* TC+TT: OR = 1.02, *P*=0.895; CC *vs.* TT: OR = 1.02, *P*=0.840; TC *vs.* TT: OR = 0.98, *P*=0.792, Table 3 and Figure 2). Identical results were found when we carried out subgroup analysis by ethnicity and source of controls (SOC) (Supplementary Table S5 and Figure 2). We did not identify any different conclusions after eliminating a study [9] which did not meet the HWE, indicating that the data arising from our meta-analysis are trustworthy and stable. The results of our sensitivity analysis indicated that our data were also stable and credible. Neither Egger’s nor Begg’s tests revealed obvious publication bias for the rs1805034 polymorphism (Supplementary Figure S1). General analysis showed that the *RANKL* gene rs2277438 polymorphism increased RA risk (G *vs*. A; OR = 1.21, *P*=0.047; GG *vs.* AG+AA; OR = 1.81, *P*=0.023; GG *vs.* AA; OR = 1.90, *P*=0.016; Supplementary Figure S2).

**Table 3:** Meta-analysis of the association between RANK, RANKL, OPG polymorphisms and RA risk

SNP	Comparison	Category	Category	Studies	OR (95% CI)	*P*-value	*P* for heterogeneity
RANK rs1805034	C vs. T	Total		3	0.99 (0.83, 1.19)	0.923	0.102
		Ethnicity	Caucasians	2	0.92 (0.64, 1.34)	0.670	0.037
			Chinese	1	1.05 (0.89, 1.24)	0.551	–
		SOC	PB	1	0.75 (0.55, 1.02)	0.066	–
			HB	2	1.07 (0.95, 1.21)	0.267	0.766
	CC+TC vs. TT	Total		3	0.99 (0.85, 1.16)	0.927	0.172
		Ethnicity	Caucasians	2	0.88 (0.70, 1.10)	0.264	0.232
			Chinese	1	1.11 (0.89, 1.38)	0.347	–
		SOC	PB	1	0.71 (0.46, 1.06)	0.111	–
			HB	2	1.05 (0.89, 1.24)	0.590	0.406
	CC vs. TC+TT	Total		3	1.02 (0.72, 1.46)	0.895	0.092
		Ethnicity	Caucasians	2	1.02 (0.54, 1.91)	0.949	0.048
			Chinese	1	0.95 (0.64,1.38)	0.780	–
		SOC	PB	1	0.70 (0.40, 1.25)	0.228	–
			HB	2	1.15 (0.82, 1.62)	0.415	0.148
	CC vs. TT	Total		3	1.02 (0.81, 1.30)	0.840	0.163
		Ethnicity	Caucasians	2	0.91 (0.47, 1.76)	0.785	0.057
			Chinese	1	1.01 (0.68,1.48)	0.975	–
		SOC	PB	1	0.62 (0.34, 1.14)	0.123	–
			HB	2	1.18 (0.87, 1.45)	0.381	0.465
	TC vs. TT	Total		3	0.98 (0.83, 1.15)	0.792	0.158
		Ethnicity	Caucasians	2	0.83 (0.65, 1.05)	0.123	0.642
			Chinese	1	1.13 (0.90,1.42)	0.280	–
		SOC	PB	1	0.75 (0.47, 1.20)	0.228	–
			HB	2	1.00 (0.76, 1.31)	0.991	0.131
RANKL rs2277438	G vs. A	Total		2	1.21 (1.00, 1.45)	0.047	0.797
	GG+AG vs. AA	Total		2	1.16 (0.93, 1.45)	0.179	0.746
	GG vs. AG+AA	Total		2	1.81 (1.09, 3.02)	0.023	0.177
	GG vs. AA	Total		2	1.90 (1.13, 3.20)	0.016	0.243
	AG vs. AA	Total		2	1.08 (0.86, 1.36)	0.515	0.492
OPG rs3102735	C vs. T	Total		5	1.22 (0.86, 1.73)	0.260	<0.001
		Ethnicity	Asians	3	1.01 (0.70, 1.46)	0.942	0.018
			Caucasians	2	1.62 (0.83, 3.13)	0.155	0.006
		SOC	HB	2	1.29 (1.04, 1.60)	0.023	0.251
			PB	3	1.16 (0.64, 2.13)	0.621	<0.001
	CC+TC vs. TT	Total		5	1.16 (0.85, 1.59)	0.338	0.004
		Ethnicity	Asians	3	0.97 (0.71, 1.33)	0.849	0.104
			Caucasians	2	1.52 (0.88, 2.61)	0.132	0.046
		SOC	HB	2	1.25 (0.98, 1.59)	0.073	0.517
			PB	3	1.11 (0.65, 1.90)	0.703	0.002
	CC vs. TC+TT	Total		5	1.73 (0.67, 4.46)	0.254	0.005
		Ethnicity	Asians	3	1.21 (0.39, 3.72)	0.745	0.036
			Caucasians	2	3.04 (0.59, 15.70)	0.183	0.050
		SOC	HB	2	2.38 (0.74, 7.66)	0.145	0.171
			PB	3	1.43 (0.34, 4.46)	0.627	0.004
	CC vs. TT	Total		5	1.79 (0.65, 4.89)	0.259	0.002
		Ethnicity	Asians	3	1.20 (0.36, 3.93)	0.766	0.026
			Caucasians	2	3.30 (0.58, 18.72)	0.177	0.039
		SOC	HB	2	2.49 (0.76, 8.20)	0.133	0.164
			PB	3	1.46 (0.31, 6.93)	0.634	0.002
	TC vs. TT	Total		5	1.03 (0.88, 1.20)	0.728	0.136
		Ethnicity	Asians	3	0.91 (0.75, 1.10)	0.337	0.389
			Caucasians	2	1.27 (0.98, 1.64)	0.067	0.320
		SOC	HB	2	1.18 (0.92, 1.52)	0.190	0.920
			PB	3	0.94 (0.77, 1.15)	0.555	0.081
OPG rs2073618	C vs. G	Total		3	1.06 (0.95, 1.19)	0.295	0.998
		Ethnicity	Asians	2	1.07 (0.92, 1.24)	0.406	0.986
			Caucasians	1	1.06 (0.89, 1.26)	0.522	–
		SOC	PB	2	1.07 (0.92, 1.24)	0.406	0.986
			HB	1	1.06 (0.89, 1.26)	0.522	–
	CC+GC vs. GG	Total			1.10 (0.94, 1.30)	0.226	0.982
		Ethnicity	Asians	2	1.10 (0.91, 1.34)	0.303	0.851
			Caucasians	1	1.10 (0.81, 1.50)	0.525	–
		SOC	PB	2	1.10 (0.91, 1.34)	0.303	0.851
			HB	1	1.10 (0.81, 1.50)	0.525	–
	CC vs. GC+GG	Total			1.04 (0.84, 1.30)	0.709	0.896
		Ethnicity	Asians	2	1.00 (0.69, 1.46)	0.982	0.687
			Caucasians	1	1.06 (0.81, 1.39)	0.659	–
		SOC	PB	2	1.00 (0.69, 1.46)	0.982	0.687
			HB	1	1.06 (0.81, 1.39)	0.659	–
	CC vs. GG	Total			1.09 (0.84, 1.42)	0.503	0.915
		Ethnicity	Asians	2	1.05 (0.71, 1.55)	0.800	0.751
			Caucasians	1	1.13 (0.79, 1.62)	0.489	–
		SOC	PB	2	1.05 (0.71, 1.55)	0.800	0.751
			HB	1	1.13 (0.79, 1.62)	0.489	–
	GC vs. GG	Total			1.11 (0.94, 1.31)	0.237	0.945
		Ethnicity	Asians	2	1.11 (0.91, 1.36)	0.286	0.750
			Caucasians	1	1.09 (0.79, 1.51)	0.600	–
		SOC	PB	2	1.11 (0.91, 1.36)	0.286	0.750
			HB	1	1.09 (0.79, 1.51)	0.600	–
OPG rs3134069	C vs. A	Total		2	0.79 (0.50, 1.25)	0.310	0.787
	CC+AC vs. AA	Total		2	0.78 (0.48, 1.26)	0.305	0.580
	CC vs. AC+AA	Total		2	0.76 (0.13, 4.52)	0.766	0.434
	CC vs. AA	Total		2	0.72 (0.12, 4.33)	0.724	0.457
	AC vs. AA	Total		2	0.78 (0.47, 1.28)	0.320	0.435

Abbreviations: HB, hospital based; PB, public based.

**Figure 3:**
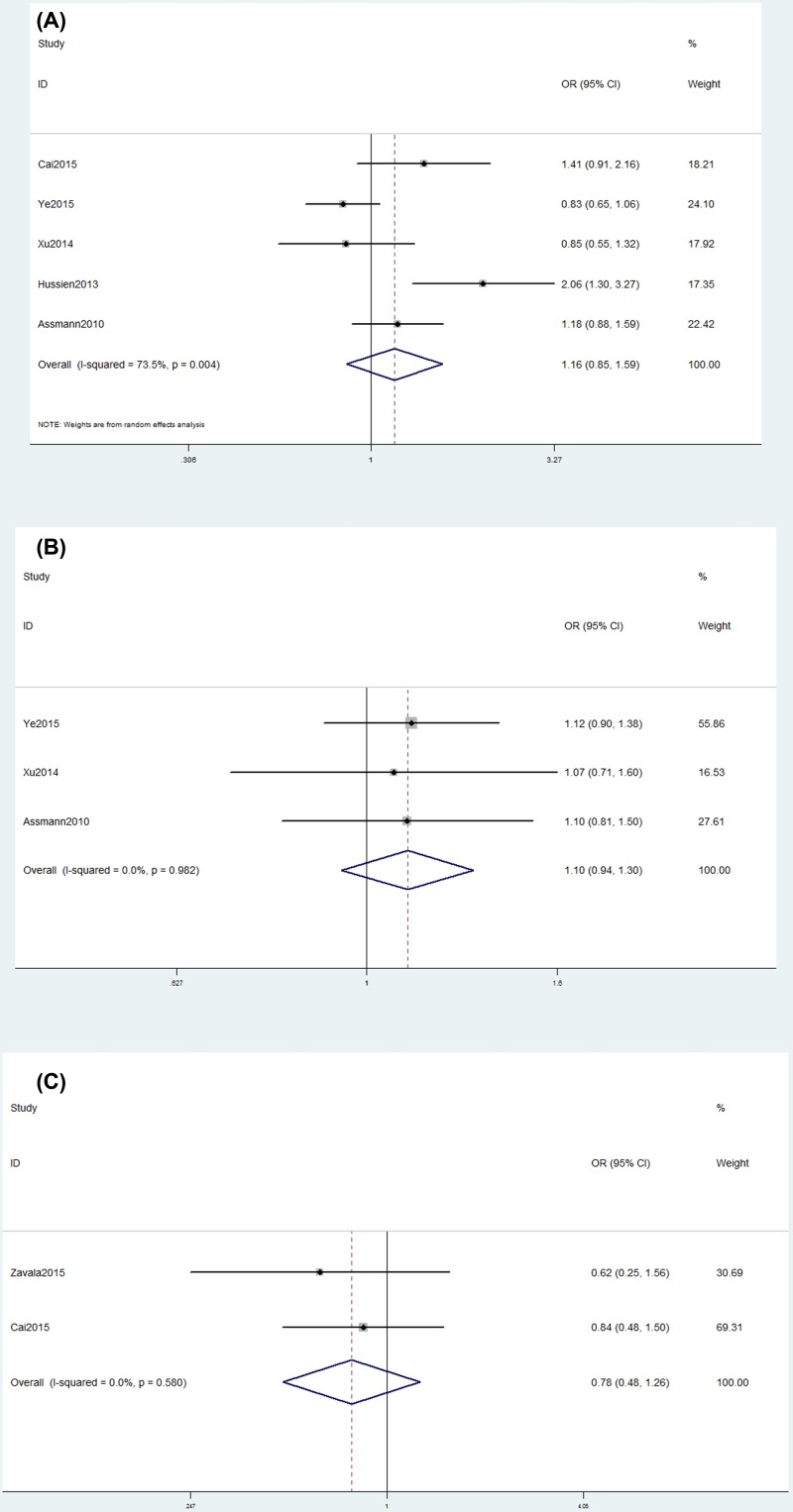
Forest plot showing the OR for associations between SNPs and RA risk (**A**) Forest plot showing the OR for associations between the rs3102735 polymorphism and RA risk (CC+TC *vs*. TT). (**B**) Forest plot showing the OR for associations between the rs2073618 polymorphism and the risk of RA (CC+GC *vs*. GG). (**C**) Forest plot showing the OR for associations between the rs3134069 polymorphism and the risk of RA (CC+AC *vs*. AA).

Pooled analysis showed that *OPG* gene rs3102735/rs2073618/rs3134069 polymorphisms were not related to RA risk (Table 3 and Figure 3). Further stratification analyses by ethnicity (Supplementary Figure S3) and SOC revealed that rs3102735/rs2073618 polymorphisms were not associated with the risk of RA among Asians or Caucasians or hospital-based and public-based studies (Table 3).

Previous research investigated rs35211496, rs7984870, rs9525641, rs9533156, rs1054016, rs531564, rs2073617 and rs3134070 polymorphisms [9,11,12,16] and reported some significant associations (Supplementary Table S6). Nevertheless, further studies are now required to confirm such associations.

## Discussion

This case-controlled study is the first study to explore the relationship between *RANK* gene rs1805034 polymorphism and the risk of RA in a Chinese Han population. The results indicated that rs1805034 polymorphism of *RANK* gene was not associated with the risk of RA. Stratification analyses by sex and age suggested that *RANK* gene rs1805034 polymorphism was not associated with the risk of RA among groups of male, female, age ≤ 55 and age > 55. This meta-analysis found that the rs2277438 polymorphism in *RANKL* gene increased the risk of RA, whereas *RANK* gene rs1805034, *OPG* gene rs3102735, *OPG* gene rs2073618, *OPG* gene rs3134069 polymorphisms were not related to RA susceptibility. Inflammatory osteoporosis is a frequent finding in RA joints and is mediated by accelerated osteoclast recruitment and activation, induced via interactions with RANK and its ligand, RANKL [19]. OPG recognizes and binds to RANKL, blocking its interaction with RANK, thus inhibiting osteoclastic differentiation and activation [20–22]. The RANK/RANKL/OPG system therefore acts as a pivotal part of the immune system and cross-links this system to bone in what has become known as osteoimmunology, a new interdisciplinary field of study integrating the disciplines of immunology and bone biology, thus providing a new perspective on the pathogenesis of RA [23–25].

Several studies have investigated the association between SNPs in *RANK* and *RANKL, OPG* genes. Assmann et al. [12] were the first to conduct such research and found that the minor allele of the *RANK* SNP rs35211496 may be protective against RA in a German population, whereas the minor alleles of the *RANKL* SNP rs2277438 may increase susceptibility to RA. In a subsequent study from China, Zhang et al. [11] revealed that there was no significant difference in the distribution of genotype or allele frequency between control subjects and RA groups. Stratification analyses by sex, age, C-reactive protein (CRP) and anti-CCP status also indicated that the *RANKL* gene rs7984870 polymorphism was not related to RA risk. Xu et al. [10] further showed that the RANKL gene rs2277438 polymorphism may not be a susceptibility factor for RA in a Chinese Han population but may have an important influence on bone and joint injury in RA. The distinct distribution of allele frequency may explain the different findings of the work carried out by Assmann et al. [12] and Xu et al. [10]. Mohamed et al. [9] observed that T allele carriers of the *RANK* gene rs1805034 polymorphism increased the risk of RA in an Egyptian population. Both Assmann et al. [12] and Mohamed et al. [9] studied the association between the *RANK* gene rs1805034 polymorphism and RA risk in Caucasian populations, but no other ethnic groups were involved. Thus, we conducted a case-controlled study in a Chinese Han population and found that the *RANK* gene rs1805034 polymorphism was not related to RA risk; this was consistent with Assmann et al. [12] but not with Mohamed et al. [9] There are several possible reasons for these different findings regarding the rs1805034 polymorphism. First, the study designs were different. The study by Assman et al. [12] study included only postmenopausal females in the RA group. Second, genetic heterogeneity for RA is known to exist in different populations (Assman et al. [12] studied a central European population while Mohamed et al. [9] studied an Egyptian population). Third, these discrepancies may be explained by clinical heterogeneity. Finally, the sample size included in the study reported by Mohamed et al. [9] was not large enough compared with the work carried out by Assmann et al. [12] and our own study, relative to Caucasian populations to support a clear conclusion. Assmann et al. [12] first reported that *OPG* gene rs3102735 polymorphism was not associated with the risk of RA. Hussien et al. [13] conducted a case-controlled study (200 cases and 150 controls) and found that *OPG* gene rs3102735 polymorphism was associated with RA susceptibility and the occurrence and development of osteoporosis in RA patients. Xu et al. [10] and Ye et al. [15] found that *OPG* gene rs3102735 polymorphism was not related to the risk of RA, whereas Cai et al. [14] reported that *OPG* gene rs3102735 polymorphism increased the risk of RA. Sample size, genetic diversity and clinical heterogeneity may explain the results of contradictions.

We realized that a single case–control study could be underpowered and inconclusive, so we carried out an additional meta-analysis together with our own case–control study. Eight published case–control studies, including 2296 cases and 2769 controls, were combined with our data to perform this meta-analysis. This represents the first meta-analysis to investigate the association between all known SNPs in *RANK/RANKL* genes and RA risk. Our meta-analysis indicated that the *RANK* gene rs1805034 polymorphism was not associated with the risk of RA, which was consistent with our own study. We also found that the *RANKL* gene rs2277438 polymorphism increased the risk of RA. To better understand the role that the RANK/RANKL/OPG network plays in the pathogenesis of RA, all reported SNPs in the *OPG* gene were also included in this meta-analysis. According to our data, *OPG* gene rs3102735, *OPG* gene rs2073618 and *OPG* gene rs3134069 polymorphisms were not related to RA susceptibility. Chen et al. [26] also performed a meta-analysis including five case-controlled studies to verify the association between these SNPs and RA risk and obtained the same results. Compared with the meta-analysis by Chen et al. [26], we consider that our meta-analysis had several additional advantages. First, our meta-analysis for the rs3134069 polymorphism included one more case-controlled study. Second, subgroup analyses were conducted by ethnicity and SOC for rs3102735 and rs2073618 polymorphisms in our meta-analysis. No significant results were found, indicating that our findings were more trustworthy.

Several potential limitations of this case–control study and meta-analysis should be considered when interpreting our results. First, we were unable to analyze subgroups of some confounding factors due to the lack of corresponding data. Second, our results were based on unadjusted estimates for confounding factors. Third, the studies included only involved Asians and Caucasians; studies among other racial groups are urgently needed. Fourth, our conclusions relating to some stratification analyses of the rs1805034 polymorphisms should be interpreted with caution, owing to limited sample size. Fifth, clinical cases should be investigated in further studies to support these analytical results. Finally, five genetic models of inheritance were used herein; thus, type I error may have arisen through a lack of correction for multiple testing.

In conclusion, this case-controlled study and meta-analysis indicated that the *RANKL* gene rs2277438 polymorphism increased the RA risk, and that *RANK* gene rs1805034, OPG gene rs3102735, OPG gene rs2073618, OPG gene rs3134069 polymorphisms were not related to RA risk. More clinical cases should now be investigated in further studies to support these analytical results.

## Supporting information

**Supplementary Figure S1:** 

**Supplementary Figure S2:** 

**Supplementary Figure S3:** 

**Supplemental Table S1:** Stratified analyses between rs1805034 polymorphism and the risk of RA.

**Supplemental Table S2:** Comparison of studied data according to RANK genotypes in all RA cases.

**Supplemental Table S3:** Comparison of studied data according to RANK genotype in all RA cases.

**Supplemental Table S4:** Comparison of studied data according to RANK genotypes in all RA cases.

**Supplemental Table S5:** The association of genetic risk score of rs1805034, rs531564 polymorphisms with risk of RA.

**Supplemental Table S6:** Characteristics of included studies

**Supplemental Table S7:** Genotype distributions of RANK, RANKL, OPG polymorphisms in the included studies NA, not available; HB, hospital-based; PB, population-based.

## Abbreviations

HWE: Hardy–Weinberg equilibrium
OPG: osteoprotegerin
OR: odds ratio
RA: rheumatoid arthritis
RANK: receptor activator of nuclear factor-κB
RANKL: RANK ligand
SNP: single nucleotide polymorphism
SOC: source of control
