# Supplementary material for: The association between RANK, RANKL and OPG gene polymorphisms and the risk of rheumatoid arthritis: a case-controlled study and meta-analysis
Source: Biosci Rep. 2019 Jun 28;39(6):BSR20182356. doi: 10.1042/BSR20182356 (PMC6597846; doi:10.1042/BSR20182356)
Supplement: Supplementary file 1 [file bsr20182356_Supp1.pdf]

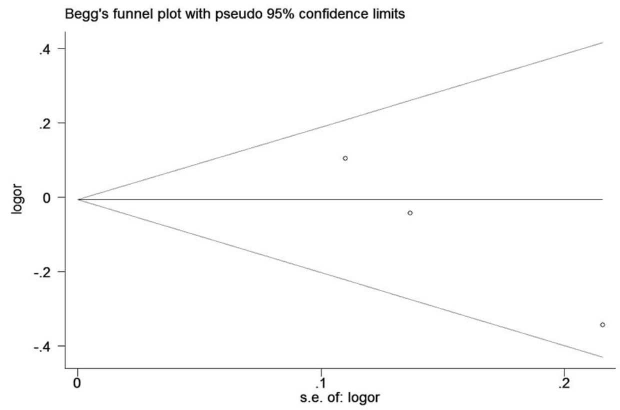
Begg's funnel plot with pseudo 95% confidence limits
.4
.2
0
-.2
-.4
logor
0
.1
.2
s.e. of: logor

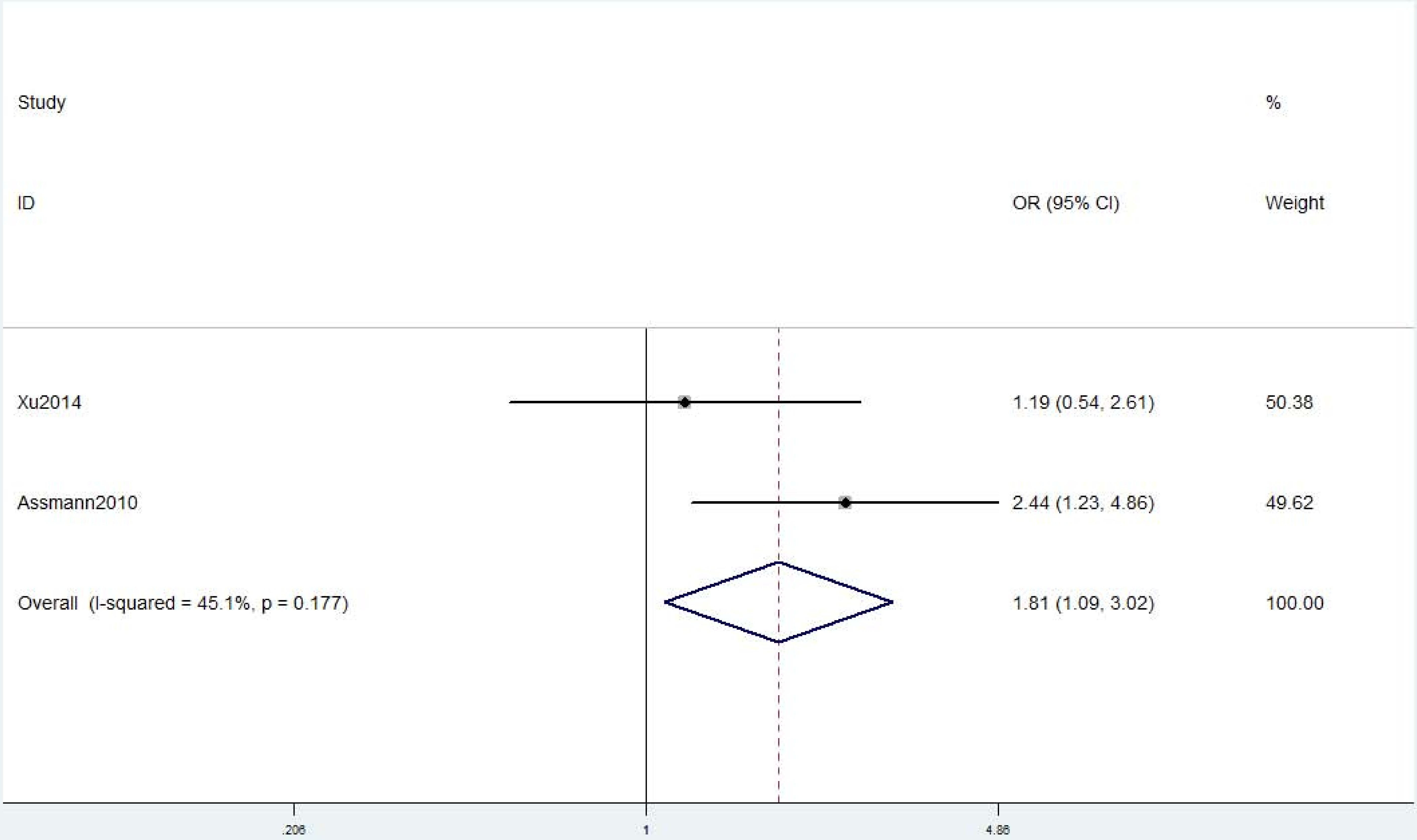
Study
%
ID
OR (95% CI)
Weight
Xu2014
1.19 (0.54, 2.61)
50.38
Assmann2010
2.44 (1.23, 4.86)
49.62
Overall (I-squared = 45.1%, p = 0.177)
1.81 (1.09, 3.02)
100.00
.206
1
4.86

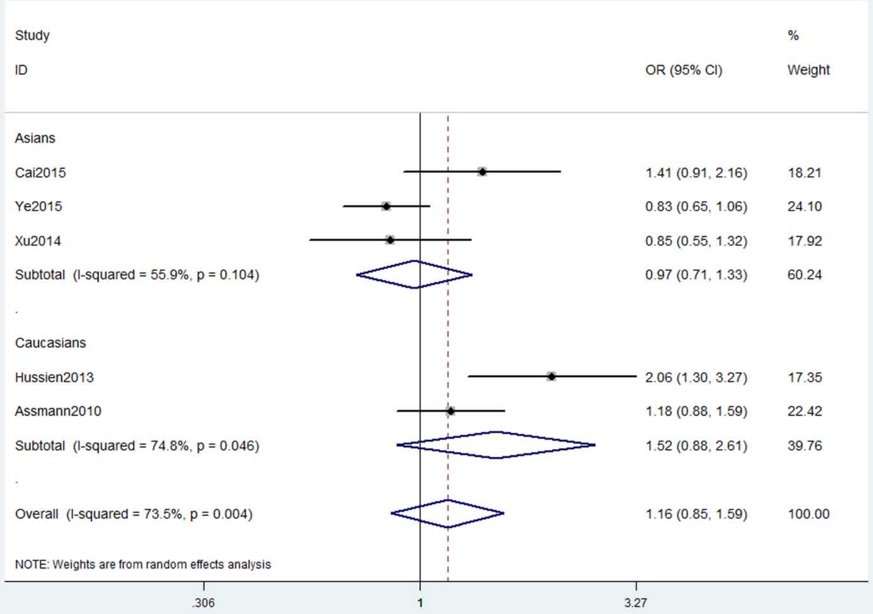
Study
ID
OR (95% CI)
%
Weight
Asians
Cai2015
1.41 (0.91, 2.16)
18.21
Ye2015
0.83 (0.65, 1.06)
24.10
Xu2014
0.85 (0.55, 1.32)
17.92
Subtotal (I-squared = 55.9%, p = 0.104)
0.97 (0.71, 1.33)
60.24
Caucasians
Hussien2013
2.06 (1.30, 3.27)
17.35
Assmann2010
1.18 (0.88, 1.59)
22.42
Subtotal (I-squared = 74.8%, p = 0.046)
1.52 (0.88, 2.61)
39.76
Overall (I-squared = 73.5%, p = 0.004)
1.16 (0.85, 1.59)
100.00
NOTE: Weights are from random effects analysis
.306
1
3.27

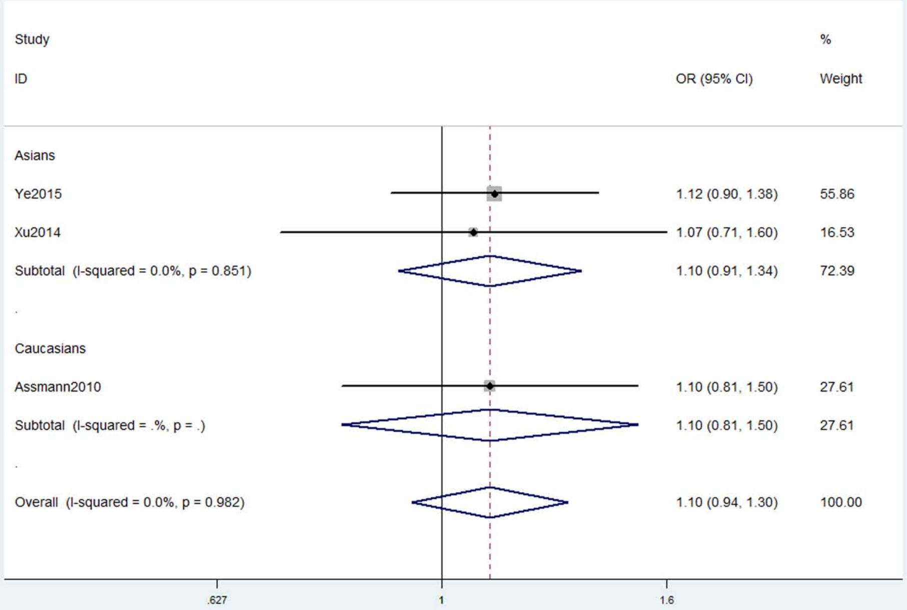
Study
ID
OR (95% CI)
%
Weight
Asians
Ye2015
1.12 (0.90, 1.38)
55.86
Xu2014
1.07 (0.71, 1.60)
16.53
Subtotal (I-squared = 0.0%, p = 0.851)
1.10 (0.91, 1.34)
72.39
Caucasians
Assmann2010
1.10 (0.81, 1.50)
27.61
Subtotal (I-squared = .%, p = .)
1.10 (0.81, 1.50)
27.61
Overall (I-squared = 0.0%, p = 0.982)
1.10 (0.94, 1.30)
100.00
.627
1
1.6

Table S1 Stratified analyses between rs1805034 polymorphism and the risk of RA

Variable	Genotype (case/control)			OR (95% CI); *P**			
rs1805034	TT	TC	CC	TC vs. TT	CC vs. TT	CC+TC vs. TT	CC vs. TC+TT
Sex							
Male	65/104	72/75	9/19	1.54(0.98,2.40); 0.060	0.76(0.32,1.78); 0.524	1.38(0.90,2.12); 0.143	0.62(0.27,1.41); 0.254
Female	201/282	181/249	43/56	1.02(0.78,1.33); 0.884	1.08(0.70,1.67); 0.737	1.03(0.80,1.32); 0.814	1.07(0.70,1.62); 0.759
Age							
≤55	129/178	136/160	31/33	1.17(0.85,1.62); 0.332	1.30(0.76,2.22); 0.346	1.19(0.88,1.62); 0.258	1.20(0.72,2.01); 0.492
＞55	137/208	117/164	21/42	1.08(0.79,1.49); 0.625	0.76(0.43,1.34); 0.340	1.02(0.75,1.38); 0.913	0.73(0.42,1.27); 0.265
rs531564	CC	CG	GG	GC vs. CC	GG vs. CC	GG+GC vs. CC	GG vs. GC+CC

OR = odds ratio; 95%CI = 95% confidence interval. *Significant *P* values if ≤ 0.05.

Table S2 Comparison of studied data according to RANK genotypes in all RA cases.

		RA(n=573)				
rs1805034		TC+CC(n=305)		TT(n=266)		*P*
Age	Years, Mean±SD	54.02±15.63		55.07±14.47		0.407
Sex	Male/Female, (n, %)	73/224	23.9/76.1	65/201	24.4/75.6	0.969
ESR	mm/h, Mean±SD	33.08±24.66		34.35±25.95		0.549
RF	Positive/Negative, (n, %)	240/65	78.7/21.3	214/52	80.5/19.5	0.603
anticcp	Positive/Negative, (n, %)	149/156	48.9/51.1	150/116	56.4/43.6	0.072
CRP	mg/L, Mean±SD	27.29±33.41		29.49±40.49		0.477
DAS28	Mean±SD	4.28±1.53		4.31±1.41		0.806
Functional class	I+II/ III+IV, (n, %)	175/130	57.4/42.6	152/114	57.1/42.9	0.955

Table S3 Comparison of studied data according to RANK genotype in all RA cases.

rs1805034		RA(n=573) CC(n=52)		TC+TT(n=519)		*P*
Age	Years, Mean±SD	51.56±17.13		54.81±14.86		0.139
Sex	Male/Female, (n, %)	9/43	17.3%/82.7%	137/382	26.4%/73.6%	0.152
ESR	mm/h, Mean±SD	32.58±27.58		33.78±25.04		0.744
RF	Positive/Negative, (n, %)	7/45	13.5%/86.5%	110/409	21.2%/78.8%	0.188
anticcp	Positive/Negative, (n, %)	24/28	46.2%/53.8%	248/271		0.822
CRP	mg/L, Mean±SD	34.51±42.11		27.69±36.28		0.204
DAS28	Mean±SD	4.10±1.36		4.31±1.49		0.317
Functional class	I+II/ III+IV, (n, %)	30/22	57.7%/42.3%	297/222	57.2%/42.7%	0.948

Table S4 Comparison of studied data according to RANK genotypes in all RA cases.

rs1805034		RA(n=573) TT (n=266)		TC (n=253)		CC (n=52)		*P*
Age	Years, Mean±SD	55.07±14.47		54.53±15.29		51.56±17.13		0.128
Sex	Male/Female, (n, %)	65/201	24.4%/75.6%	72/181	27.1%/72.9%	9/43	17.3%/82.7%	0.207
ESR	mm/h, Mean±SD	34.35±25.95		33.18±24.08		32.58±27.58		0.620
RF	Positive/Negative, (n, %)	214/52	80.5%/19.5%	195/58	77.1%/22.9%	45/7	86.5%/13.5%	0.267
anticcp	Positive/Negative, (n, %)	150/116	56.4%/43.6%	121/132	47.8%/52.2%	28/24	53.8%/46.2%	0.145
CRP	Mg/L, Mean±SD	29.49±40.49		25.80±31.22		34.51±42.11		0.130
DAS28	Mean±SD	4.31±1.41		4.32±1.56		4.10±1.36		0.364
Functional class	I+II/ III+IV, (n, %)	152/114	57.1%/42.9%	145/108	57.3%/42.7%	30/22	57.7%/42.3%	0.997

Table S5 The association of genetic risk score of rs1805034, rs531564 polymorphisms with risk of RA.

Variable	Case (n = 574)	Control (n = 804)	OR (95% CI)	*P* value	Adjusted OR (95% CI)	*P* value
	N (%)	N (%)				
First (-1.5-0)	126	241	**0.67(0.50,0.88)**	0.005	**0.66(0.49,0.87)**	0.004
Second (0)	204	260	1	-	1.0	-
Third (0-0.5)	241	270	1.14(0.88,1.46)	0.317	1.13(0.88,1.46)	0.344

The genotyping for rs1805034 and rs531564 was successful in: 571cases and 771 controls

Adjusted for sex and age

*Bold values are statistically significant ($P < 0.05$)

Table S6. Characteristics of included studies

Author	Year	Nationality	Sample size (Female/Male)		Age (mean)		Study Gene	Study SNPs	Genotype method	NOS	HWE
			case	control	case	control					
Mohamed	2016	Egypt	172 (female)	176 (female)	57.4	55.1	RANK	rs1805034	PCR-RFLP	6	N
							RANKL	rs9525641	PCR-RFLP	6	N
Xu	2014	China	201(171/30)	200(172/28)	51.9	53.5	RANKL	rs2277438	LDR-PCR	6	Y
							OPG	rs2073618	LDR-PCR	6	Y
								rs3102735	LDR-PCR	6	Y
Zhang	2013	China	329 (274/82)	697(496/201)	53.6	53.5	RANKL	rs7984870	MALDI-TOF MS	5	Y
Assmann	2010	Germany	534(392/142)	519(186/333)	49.1	34.8	RANK	rs1805034	TaqMan	5	Y
								rs35211496	TaqMan	5	Y
							RANKL	rs9533156	TaqMan	5	Y
								rs2277438	TaqMan	5	Y
								rs1054016	TaqMan	5	Y
							OPG	rs3102735	TaqMan	5	Y
								rs2073618	TaqMan	5	Y
Zavala	2015	Mexico	81(female)	52(female)	49.9	NA	OPG	rs2073617	PCR-RFLP	5	Y
								rs3134070	PCR-RFLP	5	Y
								rs3134069	PCR-RFLP	5	Y
Cai	2015	China	205(175/30)	171(97/74)	48.5	41.6	OPG	rs3102735	PCR	6	Y
								rs3134069	PCR	6	Y
Ye	2015	China	574(427/147)	804(599/205)	54.5	55.7	OPG	rs3102735	MALDI-TOF MS	6	Y
								rs2073618	MALDI-TOF MS	6	Y
Hussien	2013	Saudi Arabia	200(female)	150(female)	57.3	57.1	OPG	rs3102735	PCR-RFLP	5	Y
This study	2018	China	574(427/147)	804(599/205)	54.5	55.7	RANK	rs1805034	MALDI-TOF MS	6	Y

Table S7. Genotype distributions of RANK, RANKL, OPG polymorphisms in the included studies

NA, not available; HB, hospital-based; PB, population-based.

Gene & SNP	Author&Year	SOC	Ethnicity	Allele		CASE			CONTROL			Association with RA
				1	2	11	12	22	11	12	22	
RANK rs1805034	Mohamed2016	PB	Caucasians	T	C	87	61	24	74	69	33	Increased risk
	Assmann2010	HB	Caucasians	T	C	154	235	132	148	264	104	Not related
	This study2018	HB	Chinese	T	C	266	253	52	386	324	75	Not related
RANK rs35211496	Assmann2010	HB	Caucasians	C	T	368	128	23	330	165	21	Decreased risk
RANKL rs2277438	Xu2014	PB	Asians	A	G	95	90	15	99	77	12	Not related
	Assmann2010	HB	Caucasians	A	G	342	136	28	360	140	12	Increased risk
RANKL rs7984870	Zhang2013	HB	Chinese	C	G	90	152	78	175	344	175	Not related
RANKL rs9525641	Mohamed2016	PB	Caucasians	T	C	76	65	31	73	69	34	Not related
RANKL rs9533156	Assmann2010	HB	Caucasians	T	C	165	248	101	142	249	123	Not related
RANKL rs1054016	Assmann2010	HB	Caucasians	G	T	178	242	98	161	242	107	Not related
OPG rs3102735	Cai2015	HB	Asians	T	C	127	63	15	119	49	3	Increased risk
	Ye2015	PB	Asians	T	C	418	142	12	546	220	22	Not related
	Xu2014	PB	Asians	T	C	146	50	4	131	51	6	Not related
	Hussien2013	PB	Caucasians	T	C	116	59	25	111	36	3	Increased
	Assmann2010	HB	Caucasians	T	C	410	116	7	410	99	5	Not related
OPG rs2073618	Ye2015	PB	Asians	G	C	296	238	37	430	305	53	Not related
	Xu2014	PB	Asians	G	C	107	77	16	102	70	13	Not related
	Assmann2010	HB	Caucasians	G	C	98	276	154	103	266	143	Not related
OPG rs3134069	Zavala2015	PB	Caucasians	A	C	66	10	1	41	11	0	Not related
	Cai2015	HB	Asians	A	C	177	27	1	144	25	2	Not related
OPG rs2073617	Zavala2015	PB	Caucasians	C	T	28	26	11	9	29	13	Increased risk
OPG rs3134070	Zavala2015	PB	Caucasians	C	T	52	18	2	39	11	2	Not related
